# Deleterious effects of catecholamine administration in acute heart failure caused by unrecognized Takotsubo cardiomyopathy

**DOI:** 10.1186/s12872-018-0882-5

**Published:** 2018-07-11

**Authors:** Michael Dandel, Roland Hetzer

**Affiliations:** 1 German Centre for Heart and Circulatory Research (DZHK), Partner site Berlin, Berlin, Germany; 2 Deutsches Herzzentrum Berlin, Augustenburger Platz 1, 13353 Berlin, Germany; 3 Cardio Centrum Berlin, Berlin, Germany

**Keywords:** Acute heart failure, Catecholamines, Etiopathogenesis, Diagnosis, Myocardial infarction, Speckle-tracking echocardiography, Takotsubo cardiomyopathy

## Abstract

The potential life-threatening consequences of catecholamine use for emergency circulatory support in Takotsubo cardiomyopathy-related acute heart failure is a major challenge in cardiovascular emergences. In their recent work in BMC Cardiovascular Disorders Ansari U. et al. demonstrated the harmful effects of catecholamines on the outcome of patients with Takotsubo cardiomyopathy. Concerning this matter we emphasize the usefulness of speckle-tracking-derived echocardiography for early recognition of an acute phase of a Takotsubo syndrome in order to avoid the deleterious effects of a catecholamine therapy in patients with Takotsubo-associated acute heart failure.

## Main text

We read with great interest the publication by Ansari U. et al. [1], in which the authors report on the clinical outcomes associated with catecholamine use in patients with Takotsubo cardiomyopathy, also known as the Takotsubo syndrome (TTS). The special value of this large and comprehensive study is that it convincingly demonstrated the harmful effects of catecholamine therapy on the outcome of hemodynamically unstable patients with TTS.

Although the etiopathogenesis of TTS is still under debate, the role of catecholamines appears central to the pathophysiology of TTS. There is increasing evidence that the reversible change in left ventricular (LV) shape (apical ballooning) associated with potentially life-threatening LV dysfunction, which characterize the acute phase of TTS reflect an uncommon myocardial response to sympathetic stimulation [2–4] Patients with TTS reveal abnormal catecholamine dynamics during stress, as well as significant differences in myocardial β-adrenoreceptor (β-AR) regional distribution and sensitivity in comparison with healthy controls [2, 3]. There are also studies which suggest a genetic predisposition for altered catecholamine susceptibility and β-adrenergic signalling in TTS [3].

Catecholamines are still the first-line therapeutics in cardiogenic shock following acute myocardial infarction (MI), but are absolutely contraindicated in TTS patients, regardless of the severity of hemodynamic compromise. The potentially life-threatening consequences of catecholamine use for emergency circulatory support if the underlying cause of acute LV dysfunction is unrecognized TTS are therefore the main problem that can derive from diagnostic errors [2, 4, 5].

Clinically (including laboratory tests), electrocardiographically, and even echocardiographically using only conventional echocardiographic techniques, TTS-related acute LV disorders closely mimic acute apical MI and without coronary angiography data these two etiopathogenically different diseases are almost indistinguishable. The only helpful conventional echocardiography signs which indicate that any sympathomimetic treatment can be deleterious are the detection of mitral valve systolic anterior movement (SAM) and high LV outflow tract (LVOT) pressure gradients. However, even during acute stress-induced LV dysfunction, LVOT obstruction because of mitral valve SAM is not evident in all patients with TTS [6]. On the other hand, in the majority of TTS-related cases of life-threatening acute heart failure (HF), emergent pharmacologic or even mechanical circulatory support is necessary already before a coronary angiography becomes possible. In such situations, speckle-tracking-derived echocardiography (STE), which is currently largely available, can be particularly beneficial to avoid diagnostic errors with dangerous impact on therapeutic decision-making. The advantages of STE are its ability to differentiate between active and passive movement of ventricular wall segments, and to detect deformation (i.e. contraction) in a visually akinetic wall segment plus to evaluate components of myocardial contractile function, such as longitudinal myocardial shortening, that cannot be visually assessed. It was observed that even in severe TTS-related acute LV dysfunction with visually clearly akinitic apical anterior regions, the two-dimensional longitudinal strain pattern usually indicates myocardial viability in the visually akinetic wall segments [4, 5]. In the acute phase of TTS, STE-derived longitudinal strain recordings can show asynchrony and dyssynergy in early systole, but in end systole, similar regional longitudinal peak strain values with normal end-systolic synchrony can be observed even in functionally severely impaired ventricles [5]. In such cases, the avoidance of catecholamines as well as the early use of extracorporeal membrane oxygenation (ECMO) in clinically highly unstable patients can be life saving. According to our experience, the initial use of a ventricular assist device should be avoided as far as possible because few days of ECMO support in combination with β-blocker therapy are usually sufficient to ensure the recovery from TTS-related acute LV failure. STE also indicates that the akinetic appearance and ballooning of the LV apex might be not only a consequence of more impaired myocardial contractility in this region. Already before the routine use of STE it was postulated that the apical hypokinesia in the acute phase TTS is caused mainly by the high wall stress induced by high levels of circulating catecholamines [7]. Indeed, during catecholamine therapy of acute HF in a patient with initially unrecognized TTS, along with the aggravation of a catecholamine-induced dynamic LVOT obstruction, the systolic circumferential wall stress (σ_c_) reached up to 7 times higher value in the apex than in basal regions [4]. Such an increase in apical σ_c_ can be high enough to oppose circumferential myocardial fiber shortening which mainly generates the visible inward movement in this region and thus could explain the visually akinetic appearance of the apical regions in spite of the unequivocal prove of myocardial viability provided by the longitudinal strain data [4, 5]. Such a massive increase in apical wall stress could also explain LV ruptures that can occur in the acute phase of TTS [8]. All these insights regarding the diagnostic and therapeutic particularities of TTS allow for two important conclusions to be drawn:

1. Transthoracic echocardiography can be useful for early distinction between TTS and acute apical MI if attention is focused on regional myocardial function using the advantages of strain imaging.
2. In patients with life-threatening acute HF, before coronary angiography becomes possible, echocardiography including also STE can identify a highly probable acute phase of TTS and in such patients, if absolutely necessary, any administration of cathecholamines should be continuously monitored by echocardiography and immediately stopped if the signs for TTS become more evident.

## Abbreviations

ECMO: Extracorporeal membrane oxygenation
HF: Heart failure
LV: Left ventricle
LVOT: Left ventricular outflow tract
MI: Myocardial infarction
SAM: Systolic anterior movement
STE: Speckle-tracking-derived echocardiography
TTS: Takotsubo syndrome
β-AR: β-adrenoreceptor
σ_c_: Circumferential wall stress
